# Comparative Analysis of the Liver and Spleen Transcriptomes between Holstein and Yunnan Humped Cattle

**DOI:** 10.3390/ani9080527

**Published:** 2019-08-05

**Authors:** Yanyan Chen, Benjuan Zeng, Peng Shi, Heng Xiao, Shanyuan Chen

**Affiliations:** 1 Kunming Institute of Zoology, Chinese Academy of Sciences, Kunming 650223, China; 2 Kunming College of Life Science, University of Chinese Academy of Sciences, Kunming 650204, China; 3 School of Life Sciences, Yunnan University, Kunming 650500, China; 4 State Key Laboratory of Genetic Resources and Evolution, Kunming Institute of Zoology, Chinese Academy of Sciences, Kunming 650223, China; 5 National Demonstration Center for Experimental Life Sciences Education, Yunnan University, Kunming 650500, China

**Keywords:** differentially expressed genes, disease resistance, Yunnan humped cattle, Holstein cattle, RNA-seq

## Abstract

**Simple Summary:**

Cattle are important agricultural animals that provide essential sources of meat, milk, fertilizer for crops, clothing, and animal traction for human use, and the demand for these products has increased in recent years. There are existing differences in disease resistance between different cattle breeds. However, the genetic basis underlying disease resistance differences is poorly understood and requires further investigation. In this study, many immune- and disease-relevant genes and pathways were identified between Holstein and Yunnan humped cattle using RNA-sequencing. The novel findings regarding the genetic basis underlying disease resistance differences between zebu cattle and taurine cattle will provide a scientific basis and key technical support for disease-resistant breeding of domestic cattle, and thus have important social and economic significance.

**Abstract:**

Previous studies have shown that Yunnan humped cattle have higher disease resistance than pure taurine cattle, such as Holsteins. However, there exists limited information about the molecular genetic basis underlying disease resistance differences between them. The objective of this study was to compare differentially expressed genes (DEGs) in the liver and spleen tissues of Holstein and Yunnan humped cattle through comparative transcriptome analysis, using RNA-sequencing. In total, 1564 (647 up- and 917 down-regulated genes) and 1530 (716 up- and 814 down-regulated genes) DEGs were obtained in the liver and spleen tissues of Holstein and Yunnan humped cattle comparison groups, respectively. Gene Ontology (GO) and Kyoto Encyclopedia of Genes and Genomes (KEGG) enrichment analysis showed that the DEGs were mainly associated with the RIG-I signaling pathway, immune responses, major histocompatibility complex (MHC) class I protein complex and complement activation, human T-cell lymphotropic virus type-I (HTLV-I) infection. Some genes related to immune function, such as *C1QB*, *CD55*, *MASP2*, *C4BPA*, *MAVS*, *NOD2*, and *CD46*, were up-regulated in Yunnan humped cattle, while *C2*, *SERPING1*, *SERPINE1*, *TIRAP*, *TLR2*, and *TLR6* were down-regulated. The expression levels of 11 selected DEGs, analyzed by quantitative reverse-transcription polymerase chain reaction (RT-qPCR), were consistent with the deep sequencing results by RNA-sequencing. Our results will provide a scientific basis and key technical support for disease-resistant breeding of domestic cattle.

## 1. Introduction

Cattle consist of two main types—taurine (*Bos taurus*) and zebu (*Bos indicus*) [1,2,3]. Yunnan humped are pure zebu cattle (*Bos indicus*) [4] distributed in the tropical and subtropical region of Yunnan Province, Southwestern China. This zebu-type breed has developed several valuable traits, such as high disease resistance and good adaptability to humid and hot climatic conditions. Compared to pure taurine cattle, such as Holstein cattle (*Bos taurus*), Yunnan humped cattle are relatively resistant to several animal diseases such as theileriosis [5], tuberculosis [6], and ticks [7], while Holstein cattle exhibit higher resistance to trypanosomiasis than Yunnan humped cattle [7,8]. However, little is known about the genetic basis underlying disease resistance differences between Holstein and Yunnan humped cattle. We speculate that the zebu cattle evolved certain mechanisms to prevent disease infection.

In recent years, the phenomenon of the disease resistance differences among cattle breeds has attracted considerable interest, and various studies have shown that there are disease resistance differences between Holstein and zebu cattle [9]. Toll-like receptor genes (TLRs, *TLR1-TLR10*) have been extensively studied in cattle breeds, and the results have indicated that the nucleotide polymorphisms between Holstein and zebu cattle are different [10,11,12], and the single-nucleotide polymorphisms (SNPs) potentially are eliciting effects on susceptibility to *Mycobacterium avium* ssp. *paratuberculosis* infection in cattle breeds [13]. Additionally, previous studies have demonstrated that the diversity of many immunity-related genes (*ART4*, *CD2*, and *IL13*) is higher within zebu cattle than in Holstein cattle [14]. However, the research involved in diseases of domestic cattle is limited to some SNPs on single immunity-related genes [10,14]. Therefore, it is crucial for scientists to determine the causes of disease resistance and to identify the genes that are associated with immunity- and disease-related differences between breeds

The spleen is the largest organ in the lymphatic system and plays an important role in the immune system [15] and many immune cells, including B cells, T cells, natural killer (NK) cells, and macrophages, exist in the spleen [16]. The liver is the largest lymph-producing organ and is associated with many diseases [17]. Therefore, both spleen and liver are excellent materials for the study of differences in disease resistance between cattle breeds. Here, we used RNA-sequencing (RNA-seq) to investigate the gene expression patterns in liver and spleen transcriptomes in Holstein and Yunnan humped cattle. Differentially expressed genes (DEGs) were identified to investigate potential molecular biomarkers in the liver and spleen transcriptomes that are related to differences in disease resistance between Holstein and Yunnan humped cattle.

## 2. Material and Methods

### 2.1. Sample Collection

All of the animals mentioned in this study were approved by the Committee on Animal Research and Ethics of Yunnan University (approval number: ynucae20190011) and all the experimental methods were performed in accordance with the relevant guidelines and regulations. Ten unrelated Yunnan humped cattle (*n* = 5, experimental group) and Holstein cattle (*n* = 5, control group) were collected from Yunnan province, China. All of these individuals were of same age, unrelated, and reared under the same standard. The liver and spleen tissues were harvested within 20 min after slaughter, and all tissue samples were preserved in RNAlater solution (TIANGEN Biotech, Beijing, China). All samples were stored at 4 °C for 24 h, then frozen at −80 °C until use.

### 2.2. RNA Extraction, Transcriptome Library Construction, and Sequencing

Total RNA was extracted from liver and spleen tissues using the TRIzol total RNA extraction kit (Invitrogen Company, Shanghai, China) following the manufacturer’s instructions. RNA integrity was evaluated using the Agilent 2100 Bioanalyzer (Agilent Technologies, Santa Clara, CA, USA). Samples with RNA Integrity Numbers (RIN) ≥7 were subjected to further analysis. The libraries were constructed using TruSeq Stranded Total RNA with Ribo-Zero Gold, according to the manufacturer’s instructions, using high-quality RNA purified from liver and spleen tissues. These libraries were sequenced on the Illumina sequencing platform (HiSeqTM 2500 or other platform) and 150 bp/125 bp paired-end reads were generated. The clean reads were deposited in the National Center for Biotechnology Information (NCBI) sequence read archive (SRA) database under accession numbers SRR8712446–SRR8712465.

### 2.3. Quality Control and Reads Mapping

Clean data (clean reads) were obtained by removing reads containing adapters and ploy-N, as well as low-quality reads (reads containing only adapters and empty reads) from the raw data. At the same time, Q20, Q30, Q40, GC-content, and sequence duplication level of the clean data were calculated using Trimmomatic [18]. All the downstream analyses were based on the clean, high-quality data. The clean reads were aligned to the reference sequence of the *Bos taurus* genome (version UMD 3.1.1) using TopHat2 [19]. Additionally, this method was used to determine the position information in the reference genome or gene, and specific characteristic information of sequenced samples.

### 2.4. Differentially Expressed Gene Analysis

Numbers of reads in the RNA-seq analysis were normalized against reads per kilo base of transcripts per million (RPKM) to compute the gene expression levels [20]. The DESeq package [21] was used to identify the DEGs in liver and spleen transcriptomes between Holstein and Yunnan humped cattle. *p*-value < 0.05, false discovery rate (FDR) < 0.05 and |log2 (FoldChange)| > 2 were set as thresholds for significant differential expression.

### 2.5. Enrichment Analysis of Differentially Expressed Genes (DEGs)

To further investigate and compare the functions of those DEGs, Gene Ontology (GO) enrichment analysis and Kyoto Encyclopedia of Genes and Genomes (KEGG) pathway analysis were performed using the DAVID bioinformatics resource tool [22] to describe the biological process (BP), cellular components (CC), molecular function (MF), and KEGG pathway. GO and KEGG terms were calculated by Fisher’s exact test with a threshold *p*-value < 0.05.

### 2.6. Quantitative Reverse-Transcription Polymerase Chain Reaction (RT-qPCR) Validation

We randomly selected 11 DEGs for quantitative reverse-transcription polymerase chain reaction (RT-qPCR) validation to confirm the reliability and accuracy of the RNA-seq method. Quantification was performed with a two-step reaction process: reverse transcription (RT) and PCR. Each RT reaction had two steps. In the first step, 0.5 µg RNA and 2 µL of 4× gDNA wiper Mix were added to nuclease-free H_2_O, to a final volume of to 8 µL. Reactions were performed in a GeneAmp^®^ PCR System 9700 (Applied Biosystems, Foster city, CA, USA) for 2 min at 42 °C. In the second step, 2 µL of 5× HiScript II Q RT SuperMix Iia was added to the solution from the first step. Reactions were performed in a GeneAmp^®^ PCR System 9700 (Applied Biosystems, Foster city, CA, USA) for 10 min at 25 °C; 30 min at 50 °C, and 5 min at 85 °C. The 10 µL RT reaction mix was then diluted ten times in nuclease-free water and held at −20 °C. Real-time PCR was performed using a LightCycler^®^ 480 II Real-time PCR Instrument (Roche, Basel, Switzerland) with 10 µL PCR mixture that included 1 µL of cDNA, 5 µL of 2× QuantiFast^®^ SYBR^®^ Green PCR Master Mix (QIAGEN, Hilden, Germany), 0.2 µL of forward primer, 0.2 µL of reverse primer, and 3.6 µL of nuclease-free water. Reactions were incubated in a 384-well optical plate (Roche, Basel, Switzerland) at 95 °C for 5 min, followed by 40 cycles of 95 °C for 10 s, and 60 °C for 30 s. Each sample was run in triplicate for analysis. At the end of the PCR cycles, melting curve analysis was performed to validate the specific generation of the expected PCR product. The specific primers used in these experiments are listed in Table 1. The expression levels of mRNAs were normalized to Glyceraldehyde-3-phosphate dehydrogenase (*GAPDH*) and were calculated using the 2^−ΔΔCt^ method [23].

## 3. Results

### 3.1. RNA-Seq and Mapping

After sequencing using Illumina HiseqTM2000, 20 GB of raw reads were generated per sample library. A total of 400 GB raw reads were obtained from 20 libraries. After removal of low-quality reads (reads containing only adapters and empty reads), 753, 355, 598, and 757, 858, 666 clean reads were obtained for Yunnan humped cattle liver and spleen, respectively, and 750, 519, 172 and 775, 726, 926 clean reads were obtained for Holstein cattle liver and spleen, respectively. The Q30 scores of clean bases were more than 88.87% for all twenty tissue samples, implying the high quality of our sequencing data. We next aligned the clean reads onto the *Bos taurus* reference genome, and the mapping ratio ranged from 94.98% to 97.54%. Detailed information of RNA-seq and mapping for every sample is listed in Table 2. All samples were distributed into two groups (spleen and liver samples of both Holstein and Yunnan humped cattle) to identity the DEGs.

### 3.2. Identification of Differentially Expressed Genes

**Figure 1:**
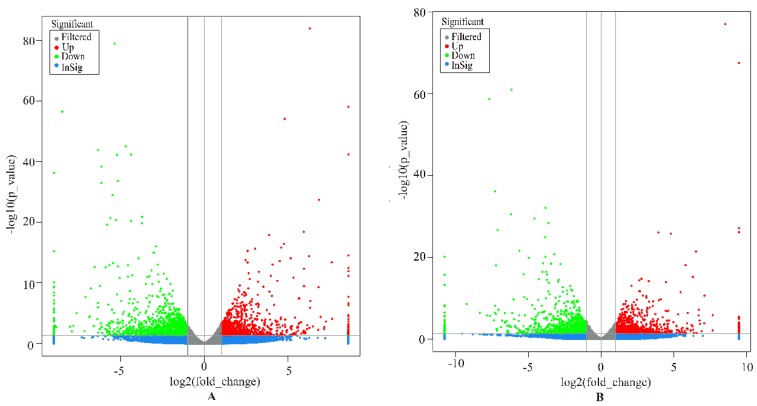
Volcano plot displaying the differentially expressed genes in the livers (**A**) and spleens (**B**) of Holstein and Yunnan humped cattle. The y-axis corresponds to the mean expression value of −log10 (*p*-value), and the x-axis displays the log2 fold-change value. The red and green dots circled in the frame represent the significant DEGs (*p* < 0.05) between Holstein and Yunnan humped cattle; the blue and grey dots represent the transcripts whose expression levels did not reach statistical significance between Holstein and Yunnan humped cattle.

**Figure 2:**
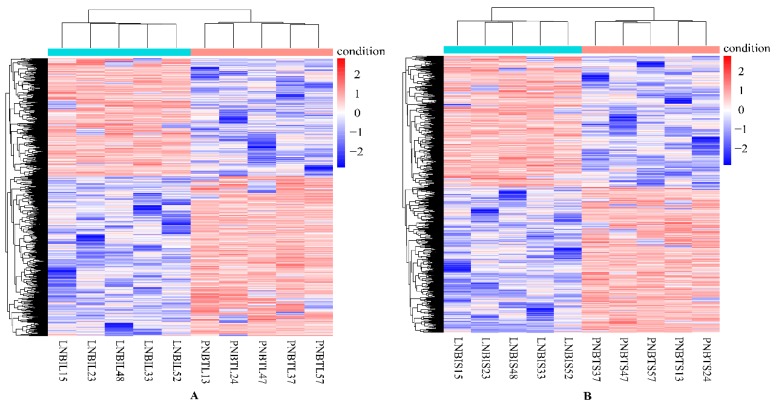
Hierarchical clustering analysis of the expression level of the DEGs in the livers (**A**) and spleens (**B**) of Holstein and Yunnan humped cattle. Red and blue indicate higher and lower expression values, respectively.

In this study, genes with FDR < 0.05, *p* < 0.05, and |log2 (FoldChange)| > 2 were considered as DEGs. Overall, a total of 1564 DEGs were identified in the liver group, with 647 up-regulated and 917 down-regulated genes (Figure 1A). In the spleen group, 1530 DEGs were detected, including 716 up-regulated and 814 down-regulated genes (Figure 1B). We analyzed the expression values of the DEGs in each sample by hierarchical clustering method. The dendrogram of clustering analysis showed that the DEGs of the two groups could separate the Yunnan humped cattle samples from the Holstein cattle completely (Figure 2A,B), implying that the expression differences of the DEGs in the two groups were significant.

### 3.3. Function Enrichment Analysis for DEGs

**Figure 3:**
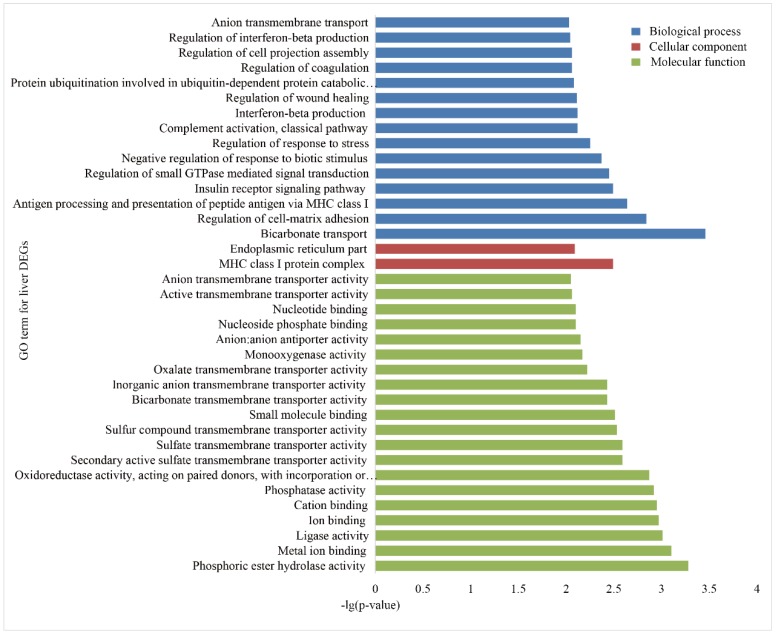
Gene Ontology (GO) enrichment analysis of the DEGs in the livers of Holstein and Yunnan humped cattle. The GO terms belonging to biological processes (BP), cellular components (CC) and molecular functions (MF) are shown in blue, red, and green, respectively. The significance levels are *p* < 0.01.

**Figure 4:**
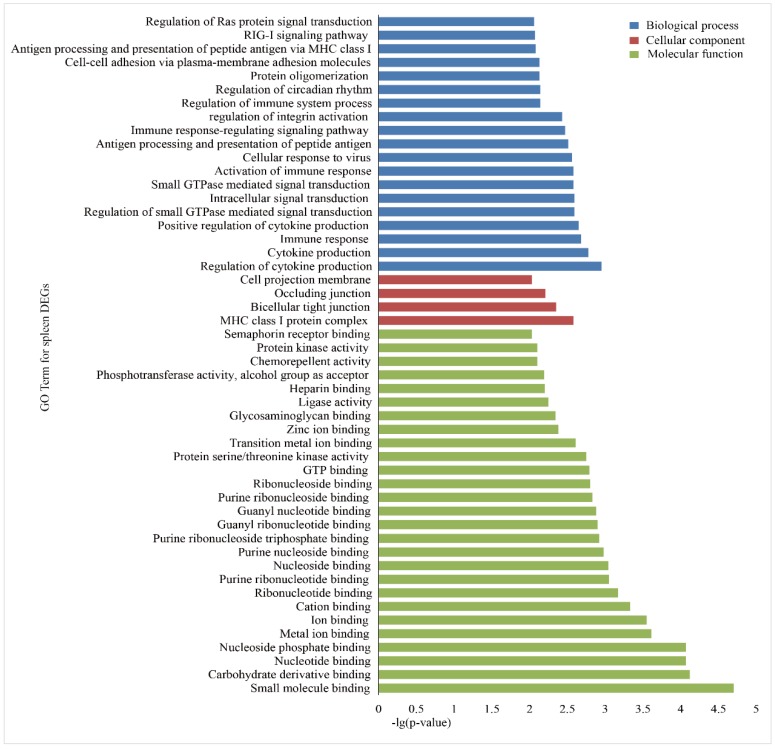
GO enrichment analysis of the DEGs in the spleens of Holstein and Yunnan humped cattle. The GO terms belonging to biological processes (BP), cellular components (CC) and molecular functions (MF) are shown in blue, red, and green, respectively. The significance levels are *p* < 0.01.

To investigate the functional association of the DEGs, GO and KEGG, enrichment analyses were performed using the database for annotation, visualization and integrated discovery (DAVID). In the liver group, 146 GO terms were significantly (*p* < 0.05) annotated within three major function groups: biological process (BP, 100), cellular component (CC, 12), and molecular function (MF, 34). The GO terms with *p*-values less than 0.01 are shown in Figure 3. The most significant GO categories observed were phosphoric ester hydrolase activity, major histocompatibility complex (MHC) class I protein complex, and metal ion binding. A total of 135 GO terms were significantly enriched in the spleen group, including 85 BP, 10 CC, and 40 MF (Figure 4), and the most significant GO categories observed were the MHC class I protein complex, which regulates cytokine production, immune responses, and activation of immune responses.

Based on KEGG pathway enrichment analysis, 11 and 15 KEGG pathways were significantly (*p* < 0.05) enriched in the liver and spleen groups, respectively. The top five pathways with the most representation of DEGs were metabolic pathways (126 DEGs), HTLV-I infection (28 DEGs), AMPK signaling pathways (15 DEGs), chemical carcinogenesis (12 DEGs), and complement and coagulation cascades (12 DEGs) in the liver group (Table 3), while metabolic pathways (113 DEGs), endocytosis (31 DEGs), the Rap1 signaling pathway (25 DEGs), phagosome (22 DEGs), and tuberculosis (21 DEGs) had the most representation in the spleen group (Table 4). Through GO and KEGG enrichment analysis, 12 and 8 immunity- and disease-related genes were identified in liver and spleen tissue in Holstein and Yunnan humped cattle (Table 5).

### 3.4. Validation Analysis Using RT-qPCR

**Figure 5:**
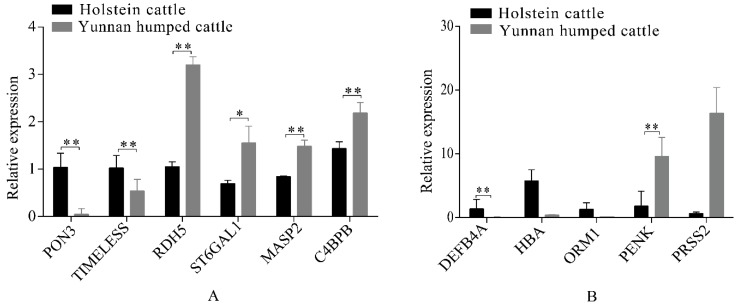
Quantitative reverse-transcription polymerase chain reaction (RT-qPCR) analysis of differentially expressed genes in the livers (**A**) and spleens (**B**) of Holstein and Yunnan humped cattle. Significance levels: * *p*-value < 0.05, ** *p*-value < 0.01.

Eleven significant DEGs, identified from the RNA-seq data, were randomly selected for RT-qPCR validation, including six genes in the liver group (*RDH5*, *ST6GAL1*, *MASP2*, *C4BPB*, *PON3*, and *MTHFS*) and five genes in the spleen group (*DEFB4A*, *HBA*, *ORM1*, *PENK*, and *PRSS2*). The RT-qPCR confirmed that the DEGs had the same pattern of expression as observed with the RNA-seq (Figure 5A,B). Therefore, gene expression observed in the liver and spleen transcriptomes of Holstein and Yunnan humped cattle was highly credible.

## 4. Discussion

In RNA-seq, RNA information of biological samples was generated from cDNA sequences using high-throughput sequencing technologies [24,25]. As an advanced technique, RNA-seq is widely applied to study the DEGs of organisms [26,27,28]. Marioni et al. (2008) demonstrated that RNA-seq and RT-qPCR have a high correlation, and that the Pearson correlation could reach 0.929 [29], which means RNA-seq is accurate and reproducible. Therefore, the objective of this study was to investigate DEGs in liver and spleen tissues between Holstein and Yunnan humped cattle, using comparative transcriptome analysis and screening of candidate genes related to immune function and disease.

As the largest lymph-producing organ in the body, the liver plays an important role in the metabolism and immune system [30,31] and is associated with many diseases [17]. In total, 1564 DEGs (*p* < 0.05) were detected in the liver tissue. GO and KEGG analysis showed that some of them were related to disease, immune function and metabolism. In the KEGG enrichment analysis, we found 12 genes that were enriched in the complement and coagulation cascades pathway, which is a proteolytic cascade in blood plasma and a mediator of innate immunity. The gene *C1QB* produced immune reactions through a series of complement cascades. Previous studies show that a C1Q deficiency could lead to lupus erythematosus and glomerulonephritis [32,33]. *CD46* and *CD55* were associated with complement and coagulation regulatory transgenes [34]. *CD46* encodes a type I membrane protein and plays a major role in complement activation. Previous studies have reported its association with several autoimmune diseases [35]. *CD55* encodes a glycoprotein involved in the regulation of the complement cascade, prevention of damage to host cells, and is associated with the progression of various cancers. Previous studies show that the expression level of the *CD55* gene is greater in individuals with gastric, colon, and breast cancer, than in non-cancerous tissues [36,37,38], and the expression was higher in Yunnan humped cattle compared to Holstein cattle. This implies that Holstein cattle might have strong resistance to gastric, colon, and breast cancer. *C2* mainly participates in apoptotic cell clearance, and its sequence variation can also be associated with lupus erythematosus [39]. *MASP2* encodes a member of the peptidase S1 family of serine proteases. Kasanmoentalib et al., (2017) proved that wild-type mice have higher cytokine levels and a greater survival rate than *MASP2*-deficient mice with pneumococcal meningitis [40]. Its expression was higher in Yunnan humped cattle compared to Holstein cattle, which implies that Yunnan humped cattle are generally not susceptible to pneumococcal meningitis. *F2* plays a role in maintaining vascular integrity during development and postnatal life. *SERPING1* is involved in the regulation of the complement cascade, and its sequence variation mainly causes hereditary angioedema [41]. *SERPINE1* is related to breast cancer, and the expression of this gene is significantly elevated in breast cancer tissues [42]; the results of the present study showed that its expression level was higher in Holstein cattle compared to Yunnan humped cattle. The genes of *C4BPA* and *C4BPB* are the binding proteins of the complement, and participate in the activation of the complement cascade [33]. Related pathways are immune response lectin-induced complement pathways and Creation of C4 and C2 activators.

The spleen is the largest lymphatic organ in the body, plays a critical role in the immune system [43], and contains many immune cells, including B cells, T cells, natural killer (NK) cells, and macrophages [16]. In the spleen tissues, 1530 DEGs were detected from which 135 GO terms and 15 KEGG pathways were annotated by GO and KEGG pathway enrichment analysis, respectively. Moreover, there were 19 biological process terms with *p*-values < 0.01, of which 11 were related to immune functions and 9 KEGG pathways were associated with disease and immune response, implying that Holstein and Yunnan humped cattle differed significantly in terms of disease resistance and immune systems.

Furthermore, a total of 157 DEGs participated in the immune-related biological processes and pathways. There were 9 DEGs that participated in more than five biological processes at the same time. *MAVS* encode an intermediary protein necessary in the virus-triggered beta interferon signaling pathway, and suppression of this gene can exacerbate the viral replication and killing of the host cells [44]. Its expression level was higher in Yunnan humped cattle compared to Holstein cattle. *NLRX1* is a member of the NLR family, and the protein of this gene is a regulator of mitochondrial antivirus responses. Its expression level is low in acute myocardial ischemia tissues [45]. *ANKRD17*, which is up-regulated in Yunnan humped cattle [46], plays a role in DNA replication and in both innate anti-viral and anti-bacterial immune pathways [47]. High-level expression of this gene can promote RIG-I-like (RLR) signaling in response to influenza and Sendai virus RNA, and was up-regulated in Yunnan humped cattle. *NOD2* is an intracellular pattern-recognition receptor and plays an important role in the immune system [48]. *TIRAP* is an adapter molecule associated with toll-like receptors and plays a crucial role in the *TLR4* signaling pathway of the immune system [49]. *TLR2* and *TLR6* are toll-like receptor genes and played a central role in the immune system, as also indicated in a previous study [50]. Among those genes, *MAVS*, *NLRX1*, *ANKRD17*, *NOD2*, and *CD46* were up-regulated in Yunnan humped cattle, while *TIRAP*, *TLR2*, and *TLR6* were down-regulated. Our results show that the disease resistance differences between Holstein and Yunnan humped cattle might be caused by the DEGs, and those DEGs are critical candidate genes involved with disease resistance in cattle breeds.

The present study provides a non-invasive method to identify the DEGs in liver and spleen between Holstein and Yunnan humped cattle using RNA-seq. In total, 1564 and 1530 DEGs were detected in the two comparison groups (liver and spleen tissues). GO and KEGG enrichment analysis showed that immunological pathways and disease pathways were present in both groups. These results provide valuable resources for biological research in breeding of domestic cattle. Our results contribute to the understanding of the mechanisms in the spleen and liver that strengthen the disease resistance of animals. In addition, our results provide fundamental information on the studies of the immunity base of Holstein and Yunnan humped cattle that could support the future development of selective breeding techniques.

## Figures and Tables

**Table 1:** Primer sequences of the differentially expressed genes (DEGs) for real-time polymerase chain reaction (PCR) analysis.

Gene	Forward Primer (5′→3′)	Reverse Primer (5′→3′)	Product Length (bp)
PON3	TGCCACCAGAGACCACTA	AGAAGAGCACCTGAGTCC	86
TIMELESS	AGAGGATGATGATGAGAGCGA	CGGATTCAAATTCACCACCTA	81
RDH5	GGTACGGGTCTCTATCGTG	CCAAAGTTTCCAGGTTTGTC	65
ST6GAL1	TTCAAGAAATCTCCTCGGAGC	TTGGATGGGAGGAACTCGTA	118
MASP2	GCTCCAGCCTGGATGTCA	TGCAGAGTAGAAGGCCTCA	80
C4BPB	TATTGTGGGCCACTGTCC	ATTCACATTCACAGGCTCC	73
DEFB4A	CATGCTGCAGGAGGTAGTAA	CAGTTTCTGACTCCGCATT	65
HBA	GACCAAAGCGGTGGAACA	AGGTCACTCAGTTCAGACAG	60
ORM1	ATGTCATCAAGTGCATAGGC	ATCCTTCTTCTCGTCAGTGT	62
PENK	ATGAGAAGAGTGGGTCGT	GCTTGAGGAAGCCACCGTA	67
PRSS2	TCCAGGGCATTGTGTCTT	TCCTGAATCCAGTCCACG	94
GAPDH	CACCCTCAAGATTGTCAGCA	GGTCATAAGTCCCTCCACGA	103

**Table 2:** Statistics and mapping results of RNA-seq data.

Sample	Raw Reads	Total Reads	Total Mapped	Multiple Mapped	Uniquely Mapped
LNBIL15	151,610,288	146,798,108	141,959,097 (96.70%)	11,387,576 (7.76%)	130,571,521 (88.95%)
LNBIL23	158,426,510	149,138,882	143,215,256 (96.03%)	11,038,440 (7.40%)	132,176,816 (88.63%)
LNBIL33	158,008,850	148,991,036	143,216,931 (96.12%)	10,108,526 (6.78%)	133,108,405 (89.34%)
LNBIL48	158,698,260	149,549,442	143,742,832 (96.12%)	10,320,099 (6.90%)	133,422,733 (89.22%)
LNBIL52	168,246,008	158,878,130	152,686,465 (96.10%)	11,676,419 (7.35%)	141,010,046 (88.75%)
LNBIS15	151,867,606	146,831,026	138,375,611 (94.24%)	9,902,596 (6.74%)	128,473,015 (87.50%)
LNBIS23	154,069,138	149,005,788	141,309,652 (94.84%)	9,514,847 (6.39%)	131,794,805 (88.45%)
LNBIS33	157,622,632	148,145,826	139,711,950 (94.31%)	8,507,598 (5.74%)	131,204,352 (88.56%)
LNBIS48	157,185,058	148,828,996	140,530,424 (94.42%)	9,373,622 (6.30%)	131,156,802 (88.13%)
LNBIS52	175,323,500	165,047,030	152,074,621 (92.14%)	10,333,303 (6.26%)	141,741,318 (85.88%)
PNBTL13	158,211,656	149,867,928	145,298,732 (96.95%)	9,247,889 (6.17%)	136,050,843 (90.78%)
PNBTL24	158,210,184	150,330,960	145,905,391 (97.06%)	10,646,629 (7.08%)	135,258,762 (89.97%)
PNBTL37	160,387,868	155,323,182	151,501,438 (97.54%)	10,344,558 (6.66%)	141,156,880 (90.88%)
PNBTL47	153,100,356	148,219,400	144,578,196 (97.54%)	11,445,184 (7.72%)	133,133,012 (89.82%)
PNBTL57	151,520,072	146,777,702	142,946,257 (97.39%)	9,632,568 (6.56%)	133,313,689 (90.83%)
PNBTS13	179,001,604	170,245,090	161,695,701 (94.98%)	13,838,064 (8.13%)	147,857,637 (86.85%)
PNBTS24	158,417,034	150,073,450	143,034,331 (95.31%)	11,076,554 (7.38%)	131,957,777 (87.93%)
PNBTS37	158,604,840	150,948,876	144,191,794 (95.52%)	6,735,375 (4.46%)	137,456,419 (91.06%)
PNBTS47	152,406,952	148,084,368	141,738,020 (95.71%)	11,052,917 (7.46%)	130,685,103 (88.25%)
PNBTS57	160,635,684	156,375,142	150,989,616 (96.56%)	12,356,517 (7.90%)	138,633,099 (88.65%)

LNBIL, Yunnan humped cattle liver; LNBIS, Yunnan humped cattle spleen; PNBTL, Holstein cattle liver; PNBTS, Holstein cattle spleen.

**Table 3:** Kyoto Encyclopedia of Genes and Genomes (KEGG) pathway analysis of the DEGs in the livers of Holstein and Yunnan humped cattle.

Pathway ID	Pathway Description	Number of DEGs	*p*-Value
bta01100	Metabolic pathways	126	0.0001
bta02010	ATP-binding cassette (ABC) transporters	9	0.0080
bta05204	Chemical carcinogenesis	12	0.0092
bta04610	Complement and coagulation cascades	12	0.0138
bta00051	Fructose and mannose metabolism	7	0.0192
bta00590	Arachidonic acid metabolism	11	0.0310
bta05166	HTLV-I infection	28	0.0383
bta04152	AMPK signalling pathway	15	0.0419
bta00830	Retinol metabolism	9	0.0448
bta04976	Bile secretion	10	0.0478
bta00982	Drug metabolism—cytochrome P450	9	0.0490

**Table 4:** KEGG pathway analysis of the DEGs in the spleens of Holstein and Yunnan humped cattle.

Pathway ID	Pathway Description	Number of DEGs	*p*-Value
bta04145	Phagosome	22	0.00600
bta01100	Metabolic pathways	113	0.00690
bta04144	Endocytosis	31	0.00750
bta04530	Tight junction	19	0.0098
bta04071	Sphingolipid signalling pathway	17	0.0126
bta04380	Osteoclast differentiation	18	0.0165
bta04015	Rap1 signalling pathway	25	0.0211
bta04514	Cell adhesion molecules (CAMs)	19	0.0295
bta04340	Hedgehog signalling pathway	6	0.0312
bta05152	Tuberculosis	21	0.0382
bta04974	Protein digestion and absorption	12	0.0394
bta04610	Complement and coagulation cascades	11	0.0400
bta05323	Rheumatoid arthritis	13	0.0411
bta04666	Fc gamma R-mediated phagocytosis	12	0.0424
bta05162	Measles	14	0.0460

**Table 5:** The DEGs related to immunity and diseases in liver and spleen tissue of Holstein and Yunnan humped cattle.

Tissue	Gene	Description	Expression Up_Down	*p*-Value
Liver	*TLR3*	Toll Like Receptor 3	Up	0.0107
	*TLR7*	Toll Like Receptor 7	Up	0.0326
	*C1QB*	Complement C1q B Chain	Up	0.0031
	*CD46*	CD46 Molecule	Up	0.0001
	*CD55*	CD55 Molecule	Up	0.0003
	*C2*	Complement C2	Down	0.0025
	*MASP2*	Mannan Binding Lectin Serine Peptidase 2	Up	0.0000
	*F2*	Coagulation Factor II, Thrombin	Up	0.0000
	*SERPING1*	Serpin Family G Member 1	Down	0.0003
	*SERPINE1*	Serpin Family E Member 1	Down	0.0005
	*C4BPA*	Complement Component 4 Binding Protein Alpha	Up	0.0025
	*C4BPB*	Complement Component 4 Binding Protein Beta	Up	0.0000
Spleen	*MAVS*	Mitochondrial Antiviral Signaling Protein	Up	0.0128
	*NLRX1*	Nucleotide binding domain and leucine-rich repeat-containing (NLR) Family Member X1	Down	0.0003
	*ANKRD17*	Ankyrin Repeat Domain 17	Up	0.0371
	*NOD2*	Nucleotide Binding Oligomerization Domain Containing 2	Up	0.0256
	*TLR2*	Toll Like Receptor 2	Down	0.0000
	*TLR6*	Toll Like Receptor 6	Down	0.0003
	*CD46*	CD46 Molecule	Up	0.0031
	*TIRAP*	TIR Domain Containing Adaptor Protein	Down	0.0242
